# Influence of new tobacco control policies and campaigns on Quitline call volume in Korea

**DOI:** 10.18332/tid/104674

**Published:** 2019-03-22

**Authors:** Jinju Park, Luu Ngoc Minh, Sang Hwa Shin, Jin-Kyoung Oh, E Hwa Yun, Duckhyung Lee, Min Kyung Lim

**Affiliations:** 1 Department of Cancer Control and Population Health, Graduate School of Cancer Science and Policy, National Cancer Center, Goyang, Republic of Korea; 2 Division of Cancer Prevention, National Cancer Control Institute, National Cancer Center, Goyang, Republic of Korea

**Keywords:** tobacco control, cessation, public policy, taxation, quitline

## Abstract

**INTRODUCTION:**

While tobacco control policies have been adopted and enforced, and anti-smoking campaigns have been conducted, the evaluation of their impact on tobacco quitting is lacking in Korea. Therefore, the effectiveness of tobacco control policies and mass media campaigns to encourage use of the Quitline were evaluated by monitoring call volume on Quitline, which has been in operation since 2006, in Korea.

**METHODS:**

Tobacco control policies and mass media campaigns, from 1 January of 2007 to 31 December of 2016, were assessed from the review of government documents and the history of law and regulation changes. The corresponding period incoming call volumes of the Quitline were assesed. The average monthly call volume, when policies and anti-smoking advertising were implemented, was compared with that of the whole year or baseline years (2007 and 2008).

**RESULTS:**

Peak call volume occurred in 2010 when the Quitline was directly promoted on television. The call volume in the month the TV campaign aired was 5.5 times higher than the average monthly call volume in the year 2010. A relatively gradual rise in call volume was found from 2013 to 2016 when the tobacco control policies and campaigns, such as Quitline number included on cigarette packs, a fear-oriented anti-tobacco campaign on mass media, and a tax increase on tobacco was implemented, were introduced sequentially. In that period, the average monthly call volume was about five times higher than in 2007 and 2008.

**CONCLUSIONS:**

Continuous efforts to contribute to tobacco control policies and campaigns by the promotion of the Quitline is a most effective approach to raise quitting attempts. Based on the Korean experience, Quitline data may be useful for assessing the impact of tobacco control policies and campaigns in Asian Pacific countries.

## INTRODUCTION

Traditional evidence-based interventions, such as price increases, complete smoking bans in all indoor areas, comprehensive advertising bans, graphic warning labels on tobacco products, and aggressive media campaigns have had great success in leading smoking cessation efforts as well as in increasing awareness of the hazards of tobacco use^1-3^.

The Quitline, available in many countries, has been one of the most effective tobacco cessation programs implemented under the World Health Organization (WHO) Framework Convention on Tobacco Control (FCTC) Article 14^3,4^, and monitoring the call volume of this service provides a way to evaluate the effectiveness of tobacco control policies and campaigns. Many countries, such as Brazil, Canada, UK and US, have reported unprecedented call volumes following the introduction of new tobacco control policies and during mass media anti-tobacco campaigns^2,4^. These reports have provided useful evidence of the effectiveness of different types of policies and campaigns in encouraging positive attitudes and behavioral changes among smokers in real-world settings.

However, such evidence is lacking in most Asian countries, even though various national tobacco control policies and campaigns have been enforced and promoted since the FCTC guidelines were introduced and the Quitline was launched as the predominant cessation program in some countries^5,6^. In Korea, policies and campaigns to control tobacco have been incrementally introduced and promoted since the FCTC was ratified in 2005, and the Quitline service has operated since 2006. However, the performances of these policies and campaigns with regard to smokers’ motivation to quit smoking have not been evaluated in terms of changes in the Quitline call volume^7^.

In this context, we investigated the effectiveness of tobacco control policies and campaigns for stimulating smoking cessation attempts by measuring the call volume of the Quitline in Korea. Additionally, we identified the types of campaigns and policies that have had the greatest influence.

## METHODS

Since the ratification of the FCTC in 2005, various tobacco control policies have been enacted and implemented and campaigns conducted in Korea. In particular, policies to minimize secondhand smoke exposure, expand mandatory smoke-free spaces, offer appropriate cessation programs, and increase taxes have been enforced and implemented. Mass media campaigns have been conducted to support these policies as well as to address the dangers of tobacco use and secondhand smoke exposure.

A nationwide toll-free Quitline is operated by the National Cancer Center (NCC) under the aegis of the Ministry of Health and Welfare (MOHW) in Korea^7^.The Quitline provides a 1-year protocol-based systematic and comprehensive telephone counseling program for registered smokers that is administered by trained counselors. Self-help materials, short message service (SMS) messages, and e-mail services are also available. Automated call systems have improved and enhanced the performance of Quitline services and have also enabled the control of call processing and the management of call data on a real-time basis.

The timing of the introduction and the major contents of policies implemented from 2007 to 2016 were determined by reviewing documents regarding the history of laws and regulatory changes. Information on the national mass media campaigns that were implemented during the same period, including the key messages and periods of public exposure, was also collected^8^.

As the main outcome measure of the present study, we counted the number of calls coming into the system from 1 January 2007, to 31 December 2016. The average monthly call volume in each year was measured and compared with the average monthly call volumes in 2007 and 2008, the first and second year after Quitline was launched.

Descriptive statistics were applied for accounting average monthly inbound calls and volume of inbound calls in the month when a popular TV show supported the Quitline and also when advertisements were aired. The average monthly call volumes during the periods when specific policies and campaigns were introduced and implemented were compared with the average monthly call volume during the entirety of the appropriate year. Data cleaning and all analysis were done in 2017.

## RESULTS

**Figure 1:**
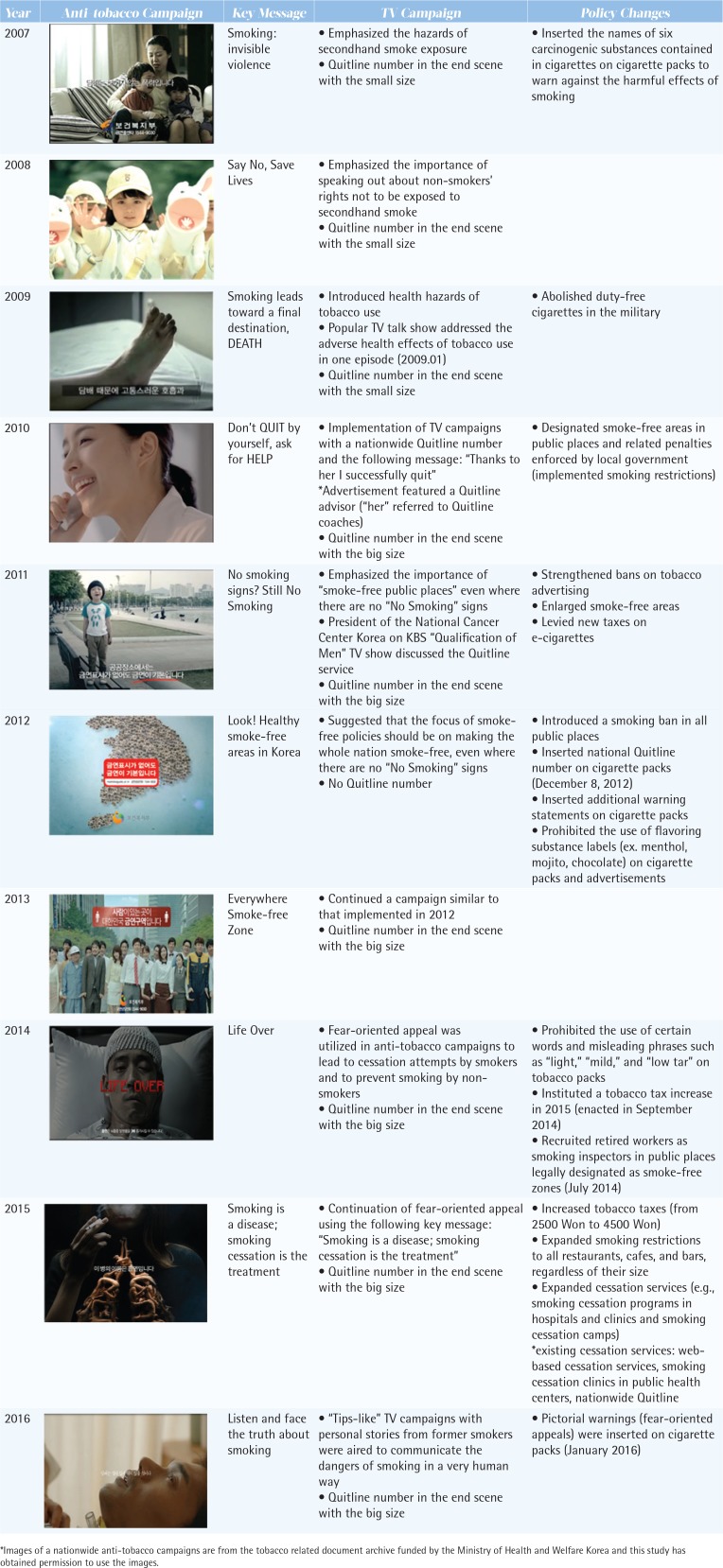
History of tobacco control policies and anti-tobacco campaigns in Korea from 2007 to 2016

In 2007 and 2008, the National Campaign for Tobacco Control, which was initiated in 2004, focused on the harmful effects of secondhand smoking. The only recognizable policy change associated with this effort was the insertion of the names of six carcinogenic substances, printed in small font, at the bottom of one side of cigarette packs. The health hazards of tobacco use and the Quitline number were introduced on a popular television talk show, and a corresponding national fear-oriented TV campaign was conducted in 2009. In 2010, outdoor smoke-free zones were designated through a regional government ordinance. From October 2010 to March 2011, the Quitline was promoted through a national TV campaign with the message: ‘Thanks to her I successfully quit’, which gave also the Quitline telephone number. In 2012, the Quitline number in the end scene of the TV campaign was stopped and instead the phrase ‘Public Health Center based Smoking Cessation Clinics’ was included, which is one of national cessation services supported by the government. Furthermore, it was legally mandated that the Quitline number appear on cigarette packs at the end of 2012. From 2011 to 2013, a national TV campaign focused on helping people understand that all venues in which humans gather should be smoke-free, on supporting smoke-free policy expansions designated by law, and on enforcing smoking bans in smoke-free places. Since 2014, there has been a national fear-oriented campaign focusing on the severe diseases caused by smoking, and a tobacco tax increase was initiated at the end of 2014 and implemented in January 2015. In 2015, additional cessation services were made available through hospitals and clinics with government support and funding through a tax increase (Figure 1).

**Figure 2:**
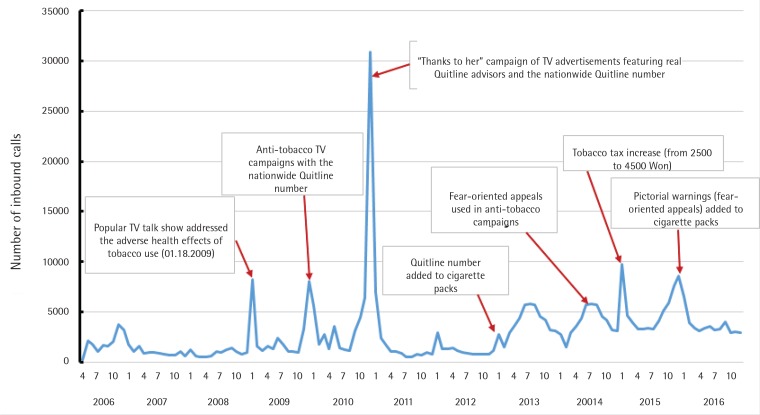
Average monthly inbound calls to the Quitline from 2006 to 2016

Although relatively short-lived, the most significant increase in call volume occurred in December 2010 when the direct promotion of the Quitline in a TV campaign was conducted from October 2010 to March 2011. The following other events may also have had an effect on increased Quitline call volume: discussion on a TV talk show (January 2009), of the adverse health effects of tobacco use; anti-tobacco campaigns including the telephone number of the Quitline (October 2009); insertion of the Quitline number on cigarette packs (December 2012); announcement by the government that a tobacco tax increase would be the next year’s tobacco control policy plan; use of fear-oriented appeals about the health hazards of tobacco use in national anti-tobacco TV campaigns (September 2014); and implementation of the tobacco tax increase (January 2015; Figure 2).

The average monthly call volume in each year from 2009 to 2015 (except 2012) was higher than the average monthly call volumes in 2007 and 2008 (Table 1). From October 2010 to March 2011, the call volumes during the months the campaign was aired directly promoting the Quitline were 5.5 and 3.1 times higher than the average monthly call volumes in 2010 and 2011, respectively. Another achievement was the relatively gradual upward trend in call volume from the early months of 2013 to the end of 2016 when the Quitline telephone number was printed on cigarette packs, the fear-oriented anti-tobacco campaign was aired, and the tax increase on tobacco was imposed. During that period, the average monthly call volumes for each year were approximately 4 and 5 times higher than those in 2007 and 2008, respectively.

**Table 1:** Change of average call volume when advertisements aired or policies implemented

*Period*	*Average monthly inbound calls*	*Relative volume of average monthly inbound calls compared to 2007−2008*	*Volume of inbound calls in the month the TV show or advertisements aired*	*Relative volume of inbound calls in the month the TV show or advertisements aired compared to the average volume of monthly inbound call*
2007−2008^a^	1089^a^			
2009	2725	2.5	8196	3.0
2010	5686	5.2	31436	5.5
2011	2472	2.3	7755	3.1
2012	1006	0.9	1644	1.6
2013	4269	3.9	-^b^	-^b^
2014	5847	5.4	8203	1.4
2015	6426	5.9	-^b^	-^b^
2016	6460	5.9	-^b^	-^b^

a Monthly averages of inbound calls for 2007 (n=1010) and 2008 (n=1168) were calculated.

b Inbound call volume increased steadily in 2013, 2015 and 2016.

## DISCUSSION

In this study, the effectiveness of tobacco control policies and mass media campaigns on encouraging use of Quitline were measured by monitoring call volume of Quitline in Korea. Previous studies have shown that tobacco control programs including mass media campaigns, such as TV advertisements, can be effective in increasing intentions to quit smoking, which is followed by adjustments in smoking behavior^9,10^. Revisions in tobacco control policies can further enhance changes in smoking behavior. Research conducted in Brazil found that taxes and price increases have great potential for stimulating cessation or reduction of tobacco consumption among young smokers and groups with a low educational level, suggesting that the magnitude of a cigarette price increase is an important predictor of intentions to quit or reduce smoking^11,12^.

Quitlines have been in operation in many countries, such as the US, Canada, Australia, New Zealand, and some European countries^6,13^. These countries have succeeded in promoting Quitlines through mass media campaigns, education, and the inclusion of the Quitline number on cigarette packs. Furthermore, the impact of tobacco control policies and campaigns on cessation attempts could be measured by monitoring the call volumes to the Quitline in these countries^2,4,14-18^. However, in Asian countries, data to evaluate the extent to which increases in use of Quitline reflect the effects of policies and campaigns are lacking. In this context, the present study provides evidence of the impact of tobacco control policies and anti-tobacco campaigns on smoking cessation intentions in terms of the call volume of the Quitline in Korea.

The Korean experience presented in the current study may provide valuable lessons about the impact of a combination of mass media advertisements and tobacco control policies on cessation intentions among smokers. Peak call volumes were observed directly after every anti-tobacco campaign and implementation of tobacco control policy. Furthermore, promotion of the Quitline number with newly adopted or revised anti-tobacco policies and the introduction of anti-tobacco campaigns were effective in increasing the use of Quitline, even though the increase in call volume varied according to the type and content of each policy or campaign. The addition of tobacco control policies to an advertisement that specifically promoted the Quitline during 2014 to 2016 might have increased calls over and above expectations, although clearly TV advertising related to call volume in a fairly predictable manner (Figure 2). From the data on the working budget of Ministry of Health and Welfare, the governmental budget for anti-tobacco campaigns on media increased by 6.4 billion KRW (Korean Won), 25.6 billion KRW and 26.6 billion KRW, in 2014, 2015 and 2016, respectively. A different call volume was also observed according to the Quitline promotion on TV advertisements and the introduction of tobacco control policies in each year: there was no promotion of the Quitline number on TV advertisements and no additions to tobacco control policies in 2012; promotion of the Quitline number on TV advertisements but no additions of tobacco control policies in 2013; promotion of the Quitline number on TV advertisements and additions of tobacco control policies in 2014 and thereafter (Figure 1). The results are consistent with the results from previous studies. Research in the UK reported that the Health Education Authority’s advertising campaign was very successful in generating calls to the Quitline^19^. Data on Australia’s National Tobacco Campaign indicated that advertising about negative health effects and Quitline modeling tended to improve call volume^20^. Studies in the US and New Zealand have also addressed the positive correlation between Quitline use and television advertising campaigns^21,22^. However, the increased call volume during anti-tobacco campaigns and implementation of tobacco control policies gradually decreased when such activities were discontinued as shown in 2012 when the TV campaigns did not include the Quitline number in the end scene of those campaigns.

Direct promotion of the Quitline through TV advertisements emphasizing the contribution of quit advisors to successful cessation attempts (tagline ‘Thanks to her’) had the strongest impact on call volume during the whole year observed, even though the general public was exposed to these ads for a relatively short period of time. Another important result was the positive effect of the inclusion of the Quitline number on cigarette packs in Korea. An identical pattern was observed in a large study that reported a significant increase in Quitline call volumes after the appearance of a Quitline number on tobacco packs in six countries, Denmark, France, Iceland, the Netherlands, Poland, and Sweden^15^. In these countries, the inclusion of the Quitline number on cigarette packs increased call volume during the first year of implementation, but the volume gradually decreased over time. Nonetheless, in Korea, the Quitline number was provided at the end of 2012, and its influence peaked in 2013 and remained relatively stable in the subsequent years. This may be because the insertion of the Quitline number on cigarette packs was followed by fear-oriented advertisements on TV that also included the Quitline number at the end of the scene. This might substantially increase the call volume, which was sustained for a longer period than when the Quitline was directly promoted in TV campaigns. Therefore, the introduction of fear-oriented anti-tobacco advertisements might be considered an effective method not only for raising awareness of the negative consequences of smoking but also for encouraging smokers to call Quitline. The increased call volume following the addition of the Quitline number on cigarette packs and the fear-oriented advertisements on TV continued with the tobacco tax increase. The increase began in September 2014, with public notification of the enactment of the law increasing the tobacco tax, and the pattern of increased call volume continued in 2015, when the law was implemented; indeed, the increased budget from the tobacco tax led to more frequent airing of smoking cessation campaigns on TV during popular viewing times. The increased call volume has continued in recent years. This indicates that the addition of various tobacco control policies and anti-tobacco campaigns, such as inserting the Quitline number on cigarette packs, implementing fear-oriented anti-tobacco campaigns, and enacting tax increases on tobacco, might be linked to a gradual upward trend in call volume.

The data on Quitline use provide a promising method with which to monitor the performance of cessation policies, programs, or campaigns in the future. The positive effects of television advertisements featuring the health hazards of tobacco use, the inclusion of the Quitline number on cigarette packs and enactment of the tax increase on tobacco products were effectively assessed by monitoring the impact of these policies and campaigns on call volume. Pictorial warnings were recently included on cigarette packs (January 2017), and a ‘Tips-like’ campaign, similar to the ‘Tips’ campaign in the US, was adopted at the end of 2016. Additionally, legally mandated smoke-free areas have been continuously expanded, and different types of national cessation services focusing on service accessibility and user convenience have been developed. Continued monitoring of call volume to the Quitline could be one way to measure the effects of these newly introduced policies and programs.

On the other hand, it is necessary to note that the gap between the rates of inbound and answered calls became more apparent during major anti-smoking events when the greatest increases in call volume were observed. The limited capacity of the Quitline has led to a low rate of live calls, especially right after every anti-tobacco campaign event. In this case, many smokers with cessation intentions could not be connected with real services to guide their cessation attempts. In Australia, Miller et al.^18^ observed a similar reduction in the proportion of calls answered during times of increased media activity due to the increased call volume and emphasized the importance of adequately meeting the demand from Quitline users. Anti-smoking campaigns or promotional activities obviously generate interest in Quitline services. Nevertheless, it can lead to an overload in calls, which should be noted as a limitation of these campaigns. Based on the results of this study, we suggest that Quitline services be enhanced during major anti-smoking campaigns to improve the live-response rate.

Korea has become well-known for advanced information technology systems, a unique strength recognized in this study. By equipping the Quitline call center with a highly developed system, call volume data have been automatically managed and monitored. The available analyzed data on Quitline use combined with the timing of major anti-smoking events aided in identifying the kinds of activities that significantly impact on call volume. This well-organized source of call-volume data can be used as an indirect measure of the effects of tobacco control policies and related campaigns.

## Limitations

The present study demonstrated that Quitline call volume was a meaningful indicator of the effectiveness of tobacco control policies and campaigns. Nevertheless, this study used only the calculated call rate, a quantitative measurement; it did not assess the content of calls, such as information delivery, which could be used as a qualitative measurement. This can be considered a limitation of our study. However, abstinence rate among the users of Quitline has been consistent during the follow-up period, including years that showed an upward trend in call volume (55% and 26%, for 30-days and one-year abstinence rate, respectively), which could be a complementary indicator to support qualitative analyses^23^. In this study, the abstinence rate was generally higher in Korea than in other countries, such as the US and Australia^18,24^. Thus, other factors not considered in this study may well have influenced the call volume of Quitline.

## CONCLUSIONS

Based on the Korean experience, we suggest that the Quitline, in addition to being an effective channel for helping smokers to quit, can also provide data with which to monitor the effects of anti-tobacco campaigns conducted and tobacco control policies implemented in Asian countries. Although direct promotion of Quitline with the telephone number given on TV campaigns had shown the strongest impact on call volume, continuous addition of tobacco control policies such as Quitline number on cigarette pack, tax increases, and fear-oriented pictorial warnings combined with the TV campaigns were more effective in increasing and sustaining call volumes of Quitline.

## CONFLICTS OF INTEREST

Authors have completed and submitted the ICMJE Form for Disclosure of Potential Conflicts of Interest and none was reported.
